# *Codonopsis pilosula* Polysaccharide Attenuates Tau Hyperphosphorylation and Cognitive Impairments in hTau Infected Mice

**DOI:** 10.3389/fnmol.2018.00437

**Published:** 2018-11-27

**Authors:** Qing Zhang, Yiyuan Xia, Hongbin Luo, Sheng Huang, Yongjun Wang, Yangping Shentu, Yacoubou Abdoul Razak Mahaman, Fang Huang, Dan Ke, Qun Wang, Rong Liu, Jian-Zhi Wang, Bin Zhang, Xiaochuan Wang

**Affiliations:** ^1^Department of Pathophysiology, School of Basic Medicine, Key Laboratory of Ministry of Education for Neurological Disorders, Tongji Medical College, Huazhong University of Science and Technology, Wuhan, China; ^2^Medical College, Hubei University for Nationalities, Enshi, China; ^3^Co-innovation Center of Neuroregeneration, Nantong University, Nantong, China; ^4^Department of Genetics and Genomic Sciences, Icahn School of Medicine at Mount Sinai, New York, NY, United States

**Keywords:** Alzheimer’s disease, *Codonopsis pilosula* polysaccharide, PP2A, tau, cognitive impairment

## Abstract

*Codonopsis pilosula* polysaccharide (CPPs), a natural products with potentially lower toxicity and better bioavailability has been used in traditional Chinese medicine for 1000s of years and a neuroprotective polysaccharide mitigates tau pathology in Alzheimer’s disease (AD) mouse model. However, whether CPPs can relieve AD pathology and cognitive defects remains poorly understood. Here we reported that CPPs remarkably increased the cell viability and PP2A activity, decreased tau phosphorylation in HEK 293/tau cells. Next, we employed an adeno-associated virus serotype 2 (AAV2)-induced expression of human full length tau (hTau) in C57/BL6 mice to mimic AD tau pathology. One month intragastric administration of CPPs significantly increased PP2A activity and reduced tau phosphorylation at Ser199, Ser202/Thr205 (AT8) and Thr231 in hippocampus of AAV2-hTau infected mice. Furthermore, behavioral tests revealed that CPPs rescued hTau overexpression induced cognitive defects while CPPs significantly increased the fEPSP slope and synaptic proteins including synaptotagmin and synaptophysin. Together, our data suggest that CPPs might prevent AD-like tau hyperphosphorylation via activation of PP2A and attenuates AD-like cognitive impairments through restoring the synaptic plasticity and synaptogenesis. In conclusion, our findings suggest that CPPs might be a potential candidate compound for the treatment of tau related diseases.

## Introduction

Alzheimer’s disease (AD) is a kind of neurodegenerative illness with progressive cognitive impairment (Reitz et al., 2011). The major pathological alterations of AD contain senile plaques comprised predominantly of amyloid-β (Aβ) peptides, and neurofibrillary tangles (NFTs) comprised of abnormally hyperphosphorylated tau (Pihlaja et al., 2008). Tau hyperphosphorylation induces dysfunction of microtubule assembly (Iqbal et al., 1986, 2010). In fact, NFTs is positively correlated with the severity of clinical dementia in AD, implicating hyperphosphorylation is a potent inducer of tau pathology (Arriagada et al., 1992). Therefore, the inhibition of tau hyperphosphorylation is one of the most promising therapeutic targets for the development of AD modifying drugs.

*Codonopsis pilosula* (CP), a well-known Chinese herbal medicine, has long time been prescribed in traditional folk medicine in China, Japan and Korea. CP has complex component, including polysaccharides, sesquiterpenes, saponins, polyphenolic glycosides, polyacetylenes, alkaloids, essential oils, and phytosteroids (Mizutani et al., 1988; Norr and Wagner, 1994; Wang et al., 1995; Wang and Wang, 1996). As the main bioactive components of CP extracts, the CP polysaccharide (CPP) is identified as a major constituent responsible for the therapeutic function of CP, such as antitumor, antimicrobial, immunoenhancing and antioxidant functions (Liu et al., 1988; Luo et al., 2007; Zong et al., 2012). It was reported that an administration of a neuroprotective polysaccharide not only upregulated neuronal activity but also induced a decrease in tau hyperphosphorylation in the brains of the 3xTg-AD mice (Makani et al., 2016). Other previous study have also shown that polysaccharides from different plants alleviate cognitive impairment and pathological alterations in AD animal models (Ho et al., 2010; Zhang et al., 2016). However, whether CPPs ameliorates AD pathological process, especially tau pathology is not investigated yet.

Wild-type human tau accumulation is a feature of sporadic AD, a condition characterized by progressive cognitive impairments (Mustroph et al., 2012). Recently, some research groups used full-length human tau (hTau) to mimic tau pathologies and behavioral deficits in rodent animals (Andorfer et al., 2003; Van der Jeugd et al., 2011). In the current study, we employed an adeno-associated virus serotype 2 (AAV2)-induced expression of hTau in C57/BL6 mice. Then, we investigated the effects of CPPs on tau pathologies and cognitive impairments in hTau transfected mice. We found that CPPs increases PP2A activity and attenuates tau hyperphosphorylation, which may be mainly through activation of PP2A. Hence, our findings implicate that CPPs upregulation of PP2A activity might be a mechanism-based therapeutic target for treating tau-related diseases including AD.

## Materials and Methods

### Plasmids, Viruses, Chemicals and Antibodies

AAV2-hTau-Mcherry was from Obio Technology (Shanghai, China) CO., Ltd. pAOV.CMV.bGlobin.hTau AAV2-8 (hTau, Titer = 6.82 × 10^12^v.g./ml), Bicinchoninic acid (BCA) protein detection kit was supplied by Pierce (Rockford, IL, United States). Reagents used for cell culture were provided by Gibco BRL (Gaithersburg, MD, United States). Antibodies employed in this study are showed in Table 1.

**Table 1:** Antibodies employed in this study.

Antibody	Specific	Type species	Dilution	Source
AT-8	Phosphorylated tau at Ser202/205	Mono- Human	1:1000 for WB	Thermo Rockford, IL United States
pS199	Phosphorylated tau at Ser199	Poly-Human, Mouse, Rat	1:1000 for WB	Invitrogen California, CA, United States
pT231	Phosphorylated tau at Thr231	Poly- Human, Rat	1:1000 for WB	Signalway Antibody College Park, MD, United States
Tau-5	Total tau	Mono- Mouse	1:1000 for WB	Millipore, Temecula, CA, United States
hT7	Purified human Tau	Mono- Human	1:1000 for WB	Thermo Rockford, IL United States
Synaptotagmin	Total synaptotagmin	Mono- Mouse	1:2000 for WB	Abcam, Cambridge, MA, United States
Synaptophysin	SYP 245-258	Poly- Human, Rat	1:1000 for WB	Abcam, Cambridge, MA, United States
PP2Ac	PP2A catalytic subunit	Poly-Human, Mouse, Rat	1:1000 for WB	Cell Signaling Technology, Danvers, MA, United States
β-actin	Total actin	Mono- Mouse	1:1000 for WB	Abcam, Cambridge, MA, United States


*Mono-, monoclonal; poly-, polyclonal; WB, Western blotting; p, phosphorylated.*

*Codonopsis pilosula* polysaccharide (purity: 92.2%) was obtained from Department of Chinese Medicine (Medical College, Hubei University for Nationalities, Enshi, China).

### Animals

Male C57/BL6 mice (8 weeks old, 23 ± 2 g) were provided by the Experimental Animal Central of Wuhan University. All the mice can take food and water freely in an air-conditioned room (22 ± 2°C, 12-h light/dark cycle). Mice were treated with daily intragastric administration of 100, 300 mg/kg of CPPs when saline served as control for 1 month. The behavior tests were performed on their active hours.

### PP2A Activity Assay

The phosphatase kit V2460 were used to measure PP2A activity in the cell extracts and brain lysates according to the manufacturer’s procedure (Promega).

### Novel Objective Recognition Test (NORT)

Novel object recognition (NOR) tests were performed in a 50 cm × 50 cm × 50 cm white acrylic box referring to Shentu’s study (Shentu et al., 2018). In brief, the mice were to habituate to the box two consecutive days without objects. The box was cleaned with 70% ethanol after each test. Next day the mice re-entered the arena from the same starting point, and the familiar A and B objects were obtained for 5 min. Twenty four hours later, the C object was replaced by the D object, and the mice still had 5 min to detect two objects. The video signal was transmitted to a computer in an adjacent room. The discrimination index was calculated by (TD-TA)/(TA + TD), (TC-TA)/(TA + TC). The identification index was calculated by TD/(TA + TD), TC /(TA + TC), TB/(TA + TB), TA/(TA + TB). TA, TB, TC, TD represented the time of mice exploring objects A, B, C, and D, respectively.

### Morris Water Maze Test

Morris Water Maze (MWM) was used to detect spatial reference memory (Morris, 1984). A circular arena (120 cm × 50 cm) filled with water (23 ± 2°C) was used for MWM. Then, an escape platform (10 × 10 × 15 cm) was placed on the tank, 1.5 cm below the water surface. A white titanium dioxide was added to the water. The walls of the test room were pasted with some pictures for permanent extra-maze cues. The trajectory of the mice was monitored by a video-tracking camera (placed 200 cm above the center of the pool surface). Latency time (s) to find the hidden platform were recorded during each trial. If the mice found the platform within 60 s, it was left on the platform for 20 s. If the mice was not able to find the platform within the time limit, it was gently placed on the platform 20 s. Probe tests were performed 1 or 48 h after acquisition (?).

### Golgi Staining

Mice were perfused with 400 mL of normal saline containing 0.5% sodium nitrite, followed by 400 mL of 4% formaldehyde solution and 200 mL Golgi fixative (5% chloral hydrate, 4% formaldehyde and 5% potassium dichromate) for 4 h in the dark. The brains were cut into 5 mm × 5 mm sections and incubated in the same Golgi fixative for 3 days, and then transferred to a silver solution containing 1% silver nitrate for another 3 days in the dark (?). The brains were serially cut into 100 μm sections using a vibrating microtome (Leica, VT1000S, Germany). Images were observed under the microscope (Nikon, Tokyo, Japan).

### Cell Culture and Transfection

HEK293/tau cells were cultured in a humidified incubator aerated with 95% air and 5% CO_2_ at 37°C. Dulbecco’s modified Eagle’s medium (Gibco, Invitrogen; Bleiswijk, Netherlands) was used to culture cells in the presence of 200 mg/mL G418 containing 10% fetal bovine serum.

Under the manufacturer’s instruction, HEK293/tau cells were seeded into 6-well plates 24 h before transfection with Lipofectamine 2000 (Invitrogen). Forty eight hours after transfection with plasmids, cells were rinsed and lysed with buffer containing 150 mM sodium chloride, 50 mM Tris-Cl, pH 8.0, 0.5% sodium deoxycholate, 0.02% sodium azide, 1% NP-40, 100 mg/mL phenylmethysulfonyl fluoride, 0.1% sodium dodecyl sulfate (SDS), and 10 mg/mL protease inhibitors (aprotinin, leupeptin, and pepstatin) followed by sonication for 5 s on ice. Supernatants were taken and added with equal volume of 2 Laemmli sample buffer after centrifugation at 12,000 *g* for 5 min at 4°C. Protein concentration was detected by BCA kit (Pierce, Rockford, IL, United States).

### Western Blotting

Brain tissue homogenates or cell lysates were boiled for 10 min. The loading proteins were electrophoresed in 10% SDS-polyacrylamide gel, and then transferred to nitrocellulose membranes (Amersham Biosciences). After blocking with 5% non-fat milk dissolved in TBS-Tween-20 for 1 h, the members were probed with primary antibody at 4°C overnight. Then the blots were detected using secondary antibodies at room temperature for 1 h and visualized using the Odyssey Infrared Imaging System (LI-COR Biosciences, Lincoln, NE, United States). Image J software (Rawak Software, Inc. Germany) was employed to quantitatively analyze the protein bands. We used 5 μg proteins to detect the level of actin, 10 μg for tau expression and 15 μg for PP2Ac (PP2A catalytic subunit).

### Cell Viability Assay

The viability of HEK293/tau cells was determined by the CCK-8 method. HEK293/tau cells were seeded at 1 × 10^6^ cells/ml in a 96-well plate and incubated at 37°C. After 24 h, the various concentrations of CPPs were added and these cells were incubated at 37°C for 48 h. Each concentration was repeated in three wells. After 48 h of treatment, the medium were replaced with 110 mL of the fresh medium, containing 10% CCK8. After half hour, the optical density was measured at 450 nm.

### Long-Term Potentiation (LTP)

The mice were anesthetized by isoflurane and the brain was quickly cut into artificial cerebrospinal fluid (aCSF) containing 119 mM NaCl, 26.2 mM NaHCO_3_, 2.5 mM KCl, 11 mM glucose,1 mM NaH_2_PO_4_, 2.5 mM CaCl_2,_ and 1.3 mM MgSO4 (pH 7.4). In ice-cold aCSF brain were sectioned into 350 μm thick slices using a vibrating microtome (Leica, VT1000S, Germany). The sections were transferred to the recovery chamber with oxidized aCSF for at least 1.5 h at room temperature.

Acute brain sections were transferred from the recovery chamber to a recording chamber and submerged in CSF. An 8 × 8 microelectrode array (Parker Technology, Beijing, China) were used to record the signals. The sections were laid down in the bottom plane (50 × 50 μm in size, with an interpolar distance of 150 μm) and kept submerged in the aCSF. The fEPSP in CA1 neurons was recorded by stimulating CA3 neurons using a MED64 system (Alpha MED Sciences, Panasonic). Baseline recordings were collected for a minimum of 30 min. Following baseline an induction protocol that evoked LTP was applied. LTP induction protocol consisted of 1 train of 100 Hz stimulus that lasted for 1 s, and the field potential response for 1 h after the tetanus was recorded. The LTP magnitude was quantified as the percentage change in the fEPSP slope (10–90%) taken during the 60 min interval after LTP induction.

### Nissl Staining

Thirty μm coronal sections were mounted on gelatin-coated slides. Then the sections were incubated in Cresyl violet for 10 min at room temperature, following with dehydration through 50, 75, 95, and 100% alcohol, cleaning in xylene and cover-slipped with neutral balsam. Images were observed using light microscope.

### Hippocampal Stereotactic Injection

The dentate gyrus (DG) is part of a brain region known as the hippocampus (part of the hippocampal formation). DG is thought to contribute to the formation of new episodic memories. Bilateral hippocampus DG zone of mice (bregma as reference: anteroposterior, 2 mm; lateral, -1.6 mm; ventral, -2.1 mm) were injected with pAOV.CMV.bGlobin.hTau AAV2 (hTau, Titer = 6.82 × 10^12^v.g./ml), Total volume = 2 μl) respectively as described previously (Zhao et al., 2006).

### Statistical Analyses

The number of mice used for the present study was five each group. We carried out Kolmogorov–Smirnov test to analyze Gaussian distributions in behavioral test, and found the data obeyed normal distribution (Supplementary Tables S1–S4). Data were expressed as means ± standard deviation (SD) and analyzed using commercial software (GraphPad Prism; GraphPad Software, Inc., La Jolla, CA, United States). Dunnett’s *t*-test was used to determine the different means among the groups. The level of significance was set at *p* < 0.05.

## Results

### CPPs Treatment Improved PP2A Activity and Reduced Tau Phosphorylation *in vitro*

**FIGURE 1:**
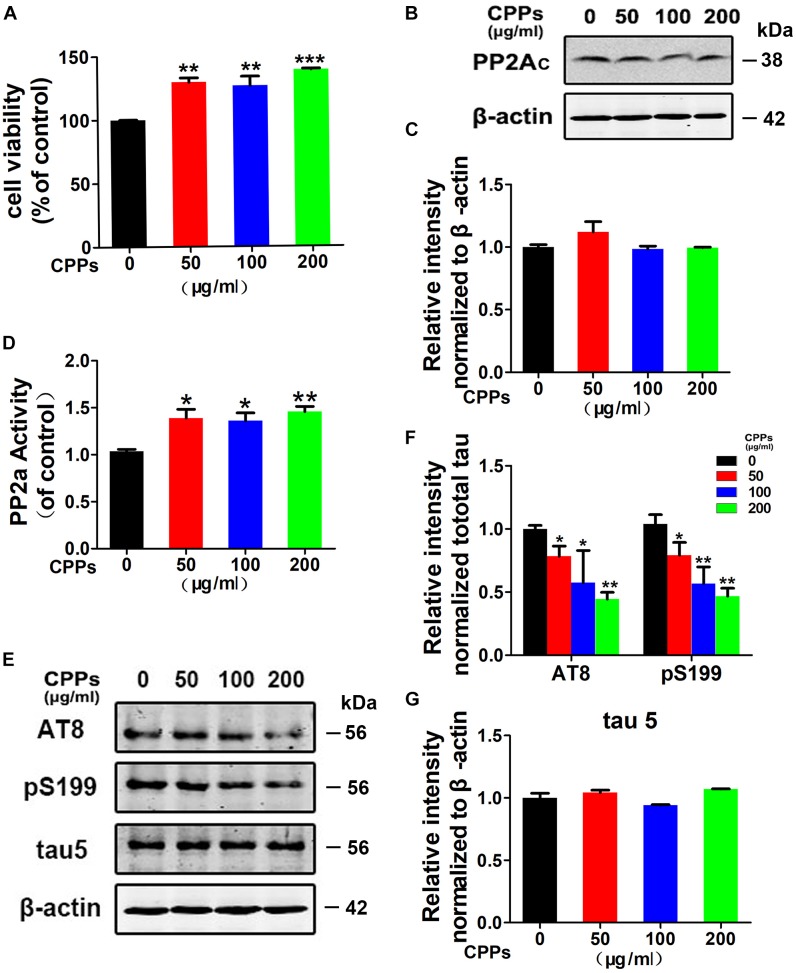
Treatment with CPPs increased PP2A activity and mitigated tau phosphorylation *in vitro*. **(A)** HEK293/tau cells were treated with CPPs for 48 h. CPPs at the indicated concentrations of 50, 100, 200 μg/ml were selected to conduct Cell Viability Assay. **(B)** The cell lysates were collected for testing the level of PP2Ac via Western blotting. **(C)** Quantitative analysis of the blots of PP2Ac. **(D)** The PP2A activity in cell lysates was measured by a chemical assay. **(E–G)** Phosphorylation levels of tau in the cells was measured by Western blotting using a panel of phosphorylation sites-specific antibodies as labeled, normalized against total tau probed by tau-5, and β-actin was used as a loading control. ^∗^*P* < 0.05, ^∗∗^*P* < 0.01, ^∗∗∗^*P* < 0.001 vs. cells without CPPs treatment.

Protein phosphatase-2A (PP2A), a major protein phosphatase in dephosphorylating tau (Liu et al., 2005), is decreased in the AD brains (Gong et al., 1993). To test the effect of CPPs on PP2A activity and tau phosphorylation, we treated HEK293/tau cells with 0, 50, 100, 200 μg/ml of CPPs. CCK-8 assay showed that all the relative survival rates in HEK293/tau cells treated with the different concentration of CPPs were significantly increased compared to control group (Figure 1A), supporting that CPPs upregulates cell viability. Next, we performed Western blots and ELISA, and found that there was no significant difference in PP2Ac level in all CPPs treated cells compared to control group (Figures 1B,C). However, the PP2A activity was significantly increased in all CPPs treated cells studied (Figure 1D). Expectedly, CPPs upregulation of PP2A activity decreased tau phosphorylation at Ser199, Ser202/Thr205 (AT8) while total tau (tau5) was not changed (Figure 1E and Supplementary Figure S1), and the quantitative data was summarized in Figures 1F,G. Together, these data suggest that CPPs treatment reduces tau phosphorylation probably through up-regulating PP2A phosphatase activity *in vitro*.

### CPPs Decreased Tau Phosphorylation in hTau Overexpression Mice

**FIGURE 2:**
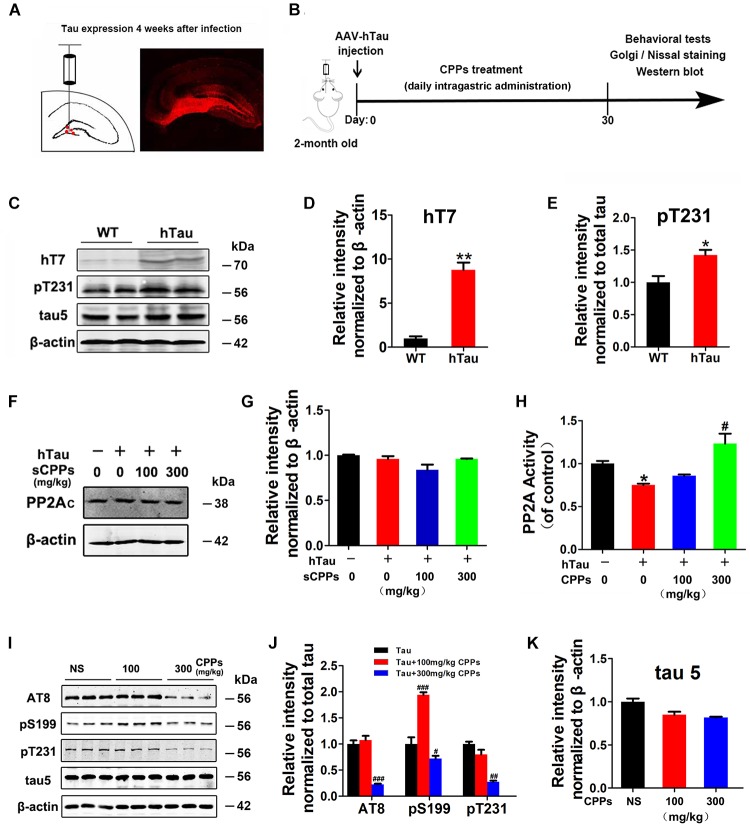
CPPs treatment mitigated tau phosphorylation in AAV2-hTau infected mice. **(A)** AAV-hTau were injected in the bilateral hippocampus DG zone of C57/BL6 mice. **(B)** Animal experimental schematics. Two-month old mice received AAV injection. Mice were treated with daily intragastric administration of 100, 300 mg/kg of CPPs when saline served as control for 1 month. After that, behavioral tests were employed and samples were collected for further biochemical studies. **(C)** After 4 weeks, the hippocampal lysates were collected for Western blots using hT7, pT231, tau5 (total tau) and β-actin. **(D,E)** Quantitative analysis of the blots. **(F,G)** hTau-overexpressed mice were treated with daily intragastric administration of 100, 300 mg/kg/day CPPs for 1 month. Relative level of PP2Ac were detected in hippocampal lysate. The lysates were collected for measurement of PP2A activity **(H)** and Western blots using AT8, pS199, pT231, tau5 (total tau) and β-actin **(I–K)**. ^∗^*P* < 0.05, ^∗∗^*P* < 0.01 vs. WT mice, #*P* < 0.05, ##*P* < 0.01, ###*P* < 0.001 vs. hTau-overexpressed mice without CPPs treatment, *n* = 4.

To further investigate whether CPPs treatment decreases tau phosphorylation *in vivo*, we injected AAV2/hTau into the hippocampus of C57/BL6 mice (Figure 2A) with daily intragastric administration of 100, 300 mg/kg of CPPs when saline served as control for 1 month (Figure 2B). We firstly carried out western blot to detect the level and phosphorylation of tau (Figure 2C), and found hTau overexpression (Figure 2D) and tau hyperphosphorylation (Figure 2E) in AAV2/hTau injected mice. Then, we investigated the effect of CPPs on PP2A activity in AAV2-hTau infected mice using ELISA and Western blots. We found that either infection with AAV2 hTau or CPPs treatment didn’t affect PP2Ac level among each groups (Figures 2F,G). However, 300 mg/kg of CPPs induced a significant increase of PP2A activity in hippocampal lysate from AAV2-hTau infected mice, but 100 mg/kg of CPPs had no effect on PP2A activity (Figure 2H). As expected, tau phosphorylation tightly responded with alteration of PP2A phosphatase activity, which acts as a major regulator of tau phosphorylation (Gong et al., 2000). We found that treatment of CPPs with 300 mg/kg not 100 mg/kg led to a remarkable decrease of tau phosphorylation at Ser199, Ser202/Thr205 (AT8) and Thr231, compared with control animals (Figures 2I,J and Supplementary Figure S2), while total tau was not altered (Figure 2K and Supplementary Figure S2). These data further indicate that CPPs increases PP2A activity and thereby decreases tau phosphorylation *in vivo*.

### CPPs Attenuates the hTau-Induced Memory Deficits

**FIGURE 3:**
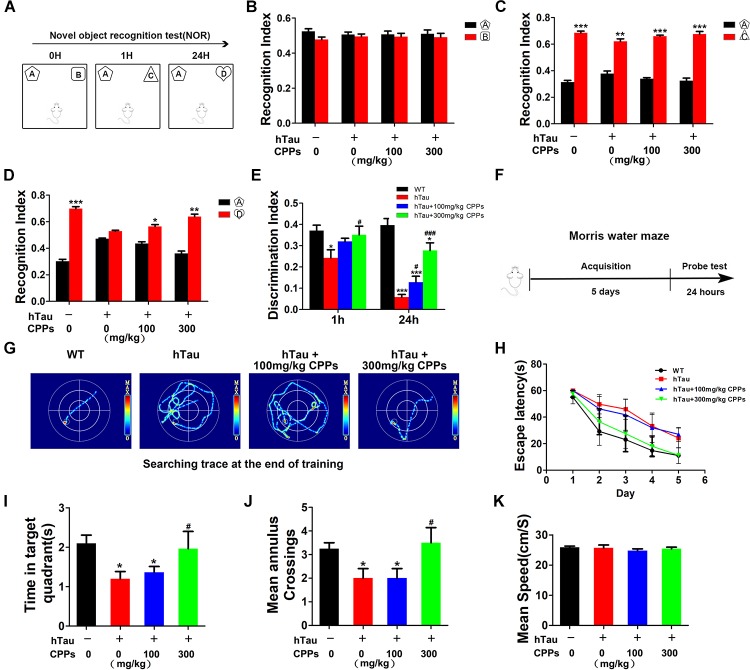
CPPs rescued hTau-induced cognitive impairments. hTau-overexpressed mice were treated with CPPs for 1 month. NOR experiment **(A)** was performed to assess the mice behavior. **(B)** Recognition index between object A and B. After 1 h, object C were used instead of object B. Recognition index were recorded in 5 min **(C)**. After 24 h, object D were used instead of object C. Recognition index between object A and D were detected **(D)**. **(E)** Discrimination index of each group. **(F,G)** Morris water maze (MWM) test were performed. **(H)** Escape latency of MWM. **(I)** The swimming time in the target quadrant in 1 min after removing the platform. **(J)** The crossings in the target quadrant in 1 min after removing the platform. **(K)** The mean swimming speed of each group. ^∗^*P* < 0.05, ^∗∗^*P* < 0.01, ^∗∗∗^*P* < 0.001 vs. WT mice, #*P* < 0.05, ###*P* < 0.001 vs. hTau-overexpressed mice without CPPs treatment, *n* = 5.

Learning and memory is a hippocampus-dependent process and hippocampal-dependant decline are the cognitive symptoms at the early stage of AD (Singh et al., 2012). To study whether CPPs rescues hTau-induced cognitive defects, we performed the NOR experiment firstly. In the acquisition trial, there was no significant difference in the recognition index among each group (Figures 3A,B). In the test trial 1 h, all mice showed comparable recognition index to the new object with increased interest (Figure 3C). Interestingly, in the test trials 24 h after the acquisition trial, a decrease in recognition index to the new objective was observed in the hTau overexpression mice (Figure 3D), indicating a long-term memory impairment. CPPs treated mice showed increased interest to the new objective compared with hTau overexpression mice (Figure 3D). The discrimination index at 1 and 24 h test trials showed the similar effects (Figure 3E). Moreover, MWM test was carried out (Figure 3F). We found that hTau overexpression led to a significant learning and memory disability compared with the control (Figures 3F–K). The mean annulus crossing and time in target quadrants were significantly reduced by the hTau injection (Figures 3I,J). However, treatment of CPPs with 300 mg/kg not 100 mg/kg restored them to control level (Figures 3I,J). Overexpression of hTau did not affect the motor activity because the swimming speed remained comparable in control and hTau overexpressed animals (Figure 3K). Thus, our data strongly support that CPPs treatment significantly prevented and rescued hTau induced learning and memory deficits.

### CPPs Enhanced Synaptic Plasticity and Functions

**FIGURE 4:**
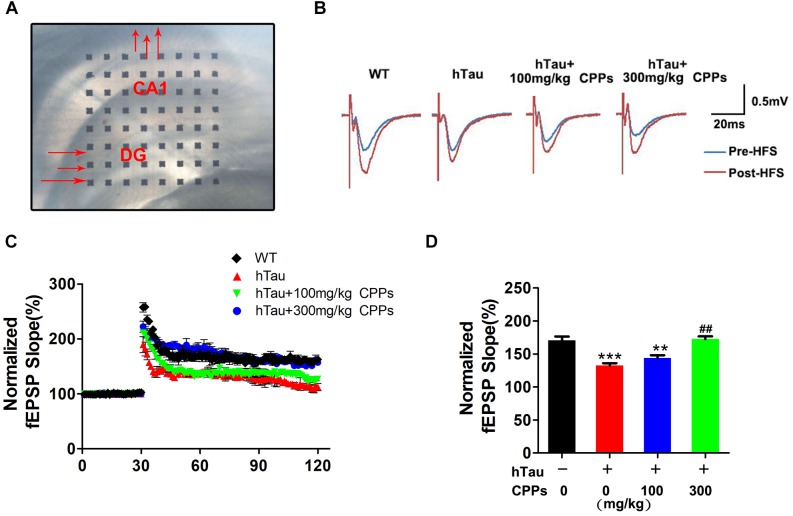
CPPs enhanced synaptic plasticity and functions. **(A)** Brain slice with MED64 array chamber in the warm artificial cerebral spinal fluid (ACSF) LTP was induced by applying three trains of high-frequency stimulation (HFS; 100 Hz, 1-s duration). **(B)** The traces are average fEPSPs before (blue) and after (red) LTP induction. **(C)** The slope of fEPSP was normalized by the baseline after HFS recorded on hippocampal slices after 1 month treated with CPPs in AVV-hTau injected mice. **(D)** Quantitative analyses for fEPSPs relative to baseline after high-frequency stimulation (HFS; 100 Hz, 1-s duration). ^∗∗^*P* < 0.01, ^∗∗∗^*P* < 0.001 vs. WT mice, ##*P* < 0.01 vs. hTau-overexpressed mice without CPPs treatment, *n* = 3.

To demonstrate indeed hTau -triggered cognitive defect and CPPs treatment were associated with synaptic plasticity deficit, electrophysiology analysis was employed (Figure 4A). We found that overexpressed hTau substantially decreased the fEPSP slope (Figures 4B–D). High dose CPPs (300 mg/kg) treatment significantly increased the fEPSP slope. In contrast, low dose CPPs (100 mg/kg) treatment didn’t display any effect compared with hTau overexpression mice (Figures 4B–D). These data suggest that hTau amplification damaged or sabotaged the synaptic plasticity and high dose CPPs treatment attenuated the loss of the synaptic plasticity.

### CPPs Recovered a Panel of Synaptic Proteins in AAV- hTau Injected Mice

**FIGURE 5:**
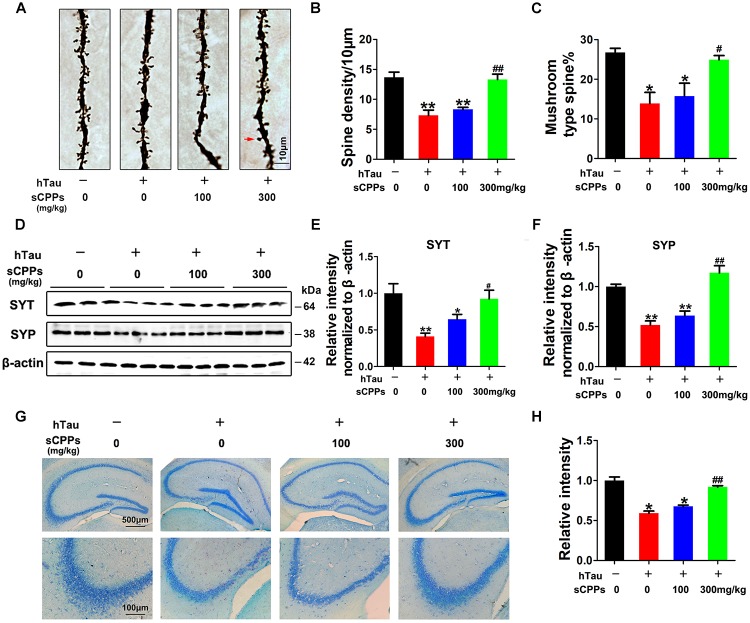
CPPs recovered memory-related proteins levels and neuronal survival. AAV-hTau was injected for 4 weeks with or without simultaneous treatment of CPPs. **(A)** The representative Golgi staining (red arrow: mushroom synapses). Scale bar = 10 μm. **(B,C)** The quantification of spine density and mushroom type spine. **(D)** Levels of synaptotagmin (SYT), and synaptophysin (SYP) were detected by western blotting in the hippocampus and β-actin was used as loading control. **(E,F)** Quantitative analysis of the blots **(G)** Nissl staining (upper row scale bar = 500 μm, lower row scale bar = 100 μm) and **(H)** the quantification of neuron density. The data were expressed as mean ± SD, ^∗^*P* < 0.05, ^∗∗^*P* < 0.01 vs. WT mice, #*P* < 0.05, ##*P* < 0.01 vs. hTau-overexpressed mice without CPPs treatment, *n* = 3.

To further investigate whether hTau overexpression and CPPs treatment affect the synapses, we carried out Golgi staining. Noticeably, dendritic spines and mushroom type spines in AAV2-hTau infected mice were significantly decreased compared to control mice (Figures 5A–C). However, treatment of CPPs with 300 mg/kg not 100 mg/kg restored dendritic spines and mushroom type spines to control level (Figures 5A–C), supporting that CPPs blocks hTau-induced cognitive defect through the restoration of synaptic plasticity.

To further prove this observation, synaptic proteins were investigated in all groups. We found that synaptic proteins including synaptotagmin and synaptophysin were markedly decreased after AAV2-hTau infection (Figures 5D–F). The administration of CPPs, as curative doses, significantly increased synaptotagmin and synaptophysin to levels comparable with the control and the quantitative data were summarized in Figures 5D–F. We also detected the total cell numbers by Nissl staining. The results showed that AAV-hTau injection significantly reduced the cell numbers in hippocampus, and treatment of CPPs reduced the cell loss (Figures 5G,H). Thus, our data strongly support that CPPs treatment had a potential pharmacological interference in restoring the synaptic plasticity and synaptogenesis.

## Discussion

Alzheimer’s disease is one of chronic and neurodegenerative disorders that is histopathologically characterized by Aβ plaques and NFTs, which lead a progressive deterioration of cognition and behavior (Janus et al., 2001). AD is becoming a public health problem at present. Over 47 million people in the worldwide are suffering from AD. By 2050, the number of AD cases could reach over 131 million in the world^1^, unless drugs are available that can prevent or cure the disease. Because NFTs mostly consist of hyperphosphorylated tau and is a key pathological event in AD (Yagami, 2006), inhibition of tau hyperphosphorylation has been regarded as one of the most promising therapeutic strategy. As previously reported, we induced AD-like pathology by AAV2-hTau infection with C57/BL6 mice in order to study the effect of CPP, with immunoenhancing, antioxidant and neuroprotective functions (Liu et al., 2015; Makani et al., 2016). We found in the current study, that CPPs recovers PP2A activity and attenuates tau phosphorylation in AAV2-hTau infected mice. Most importantly CPPs rescues hTau-induced cognitive impairment through the restoration of synaptic plasticity.

Previous study showed that intranasal administration of a neuroprotective polysaccharide decreased in the brains of 3 × Tg AD mice (Makani et al., 2016). We found CPPs treatment up-regulates cell viability and PP2A activity, reduces tau phosphorylation in HEK293/tau cells, which is probably due to activation of PP2A.

Tau is the major microtubule-associated protein, which main functions are to promote the assembly of tubulin into microtubules and to stabilize microtubule structure (Cleveland et al., 1977). Abnormally hyperphosphorylated tau fails to bind to tubulin (Iqbal et al., 1986; Alonso et al., 1994) and form NFTs (Shively et al., 2012). Therefore, abnormal hyperphosphorylation of tau has been considered as one of crucial events to neurofibrillary degeneration in AD and other tauopathies (Gong et al., 2010). By overexpression of hTau to mimic tau pathology in C57/BL6 mice, we observed a marked increase of tau phosphorylation and a decrease of PP2A activity. However, CPPs treatment increased PP2A activity and decreases tau phosphorylation in the brain of the hTau infected mice, further supporting CPPs up-regulating PP2A activity and consequently attenuating tau phosphorylation. The regulation of PP2Ac activity involves (i) interaction with regulatory B-subunit, (ii) interaction with inhibitor 1 of PP2A (I_1_^PP2A^) and inhibitor 2 of PP2A (I_2_^PP2A^), (iii) phosphorylation of PP2Ac at Y307 and (iv) methylation of PP2Ac at L309. The mechanism underlying CPPs up-regulation of PP2A activity is expected to further investigate on phosphorylation and methylation of PP2Ac, and low-energy binding conformations of CPPs bound to PP2Ac by virtual ligand docking. Interestingly, we found an increase of tau phosphorylation on PS199 with 100 mg/kg dose of CPPs while the PP2A activity had no change. We speculated that low dose of CPPs induces activation of some tau Ser 199 related kinases, which doesn’t result in cognitive impairments.

Cognitive decline is typical clinical symptoms in AD patients. In accordance with previous study (Hwangbo et al., 2012), we also found that overexpression of hTau induced a significant learning and memory disability. Treatment with CPPs recues the hTau-induced cognitive dysfunction. These results are consistent with previous study in which polysaccharides from pleurotus ostreatus attenuate cognitive defect in an AD model induced by D-galactose and AlCl3 (Zhang et al., 2016). To study the effect of CPPs on cognition, we detect synaptic plasticity. In current study, electrophysiology analysis showed that CPPs blocked the decrease in fEPSP slope induced by hTau overexpression, and Golgi staining indicate that treatment of CPPs restored dendritic spines and mushroom type spines to control level, supporting that CPPs attenuates hTau-induced cognitive defect through the restoration of synaptic plasticity. Synaptic transmission in the nerve system involves coherent presynaptic and postsynaptic actions, synaptotagmin and synaptophysin are important proteins in synaptic functions (Li and Cooper, 2001). We found that CPPs blocked the decrease in levels of synaptotagmin and synaptophysin induced by hTau expression. Consistent with previous reports (DeVos et al., 2017), our data also supported that hTau induced neuronal loss. However, CPPs treatment rescued the neuronal loss.

Taken together, we report that CPPs attenuates tau hyperphosphorylation via upregulation of PP2A activity and rescues hTau-induced behavioral deficits through the restoration of synaptic plasticity. Further studies are expected to investigate whether some molecules in the CPPs have the ability to pass through the blood brain barrier with a direct effect. This study supports that as a natural plant chemical, CPPs might be promising therapeutic candidate for AD.

## Ethics Statement

All animal experiments were approved by the Animal Care and Use Committee of Huazhong University of Science and Technology, and performed in compliance with the National Institutes of Health Guide for the Care and Use of Laboratory Animals.

## Author Contributions

XW designed the research. QZ, YX, SH, YW, YM, FH, YS, DK, and QW performed the research. RL, J-ZW, HL, BZ, and XW analyzed the data. XW wrote the paper.

## Conflict of Interest Statement

The authors declare that the research was conducted in the absence of any commercial or financial relationships that could be construed as a potential conflict of interest.

## Footnotes

1 https://www.alz.co.uk/research/WorldAlzheimerReport2016.pdf

## Supplementary Material

FIGURE S1 The level and phosphorylation of tau in HEK 293/tau cells. Levels of total tau **(A)**, phosphorylated tau at Ser199 **(B)** and Ser202/Thr205 (AT8) **(C)** were detected by western blotting in HEK293/tau cells.

FIGURE S2 The level and phosphorylation tau in AAV2-hTau infected mice. Levels of total tau **(A)**, phosphorylated tau at Ser199 **(B)**, Thr231 **(C),** and Ser202/Thr205 (AT8) **(D)** were detected by western blotting in AAV2-hTau infected mice.

TABLE S1 A recognition index.

TABLE S2 B recognition index.

TABLE S3 D recognition index.

TABLE S4 Discrimination index-24H.
