# Supplementary figures and images for: Codonopsis pilosula Polysaccharide Attenuates Tau Hyperphosphorylation and Cognitive Impairments in hTau Infected Mice
Source: Front Mol Neurosci. 2018 Nov 27;11:437. doi: 10.3389/fnmol.2018.00437 (PMC6277749; doi:10.3389/fnmol.2018.00437)

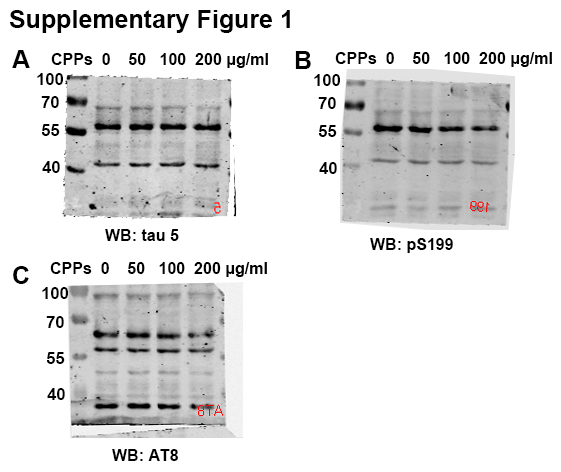

Supplement: FIGURE S1 — The level and phosphorylation of tau in HEK 293/tau cells. Levels of total tau (A), phosphorylated tau at Ser199 (B) and Ser202/Thr205 (AT8) (C) were detected by western blotting in HEK293/tau cells. [file Image_1.JPEG]

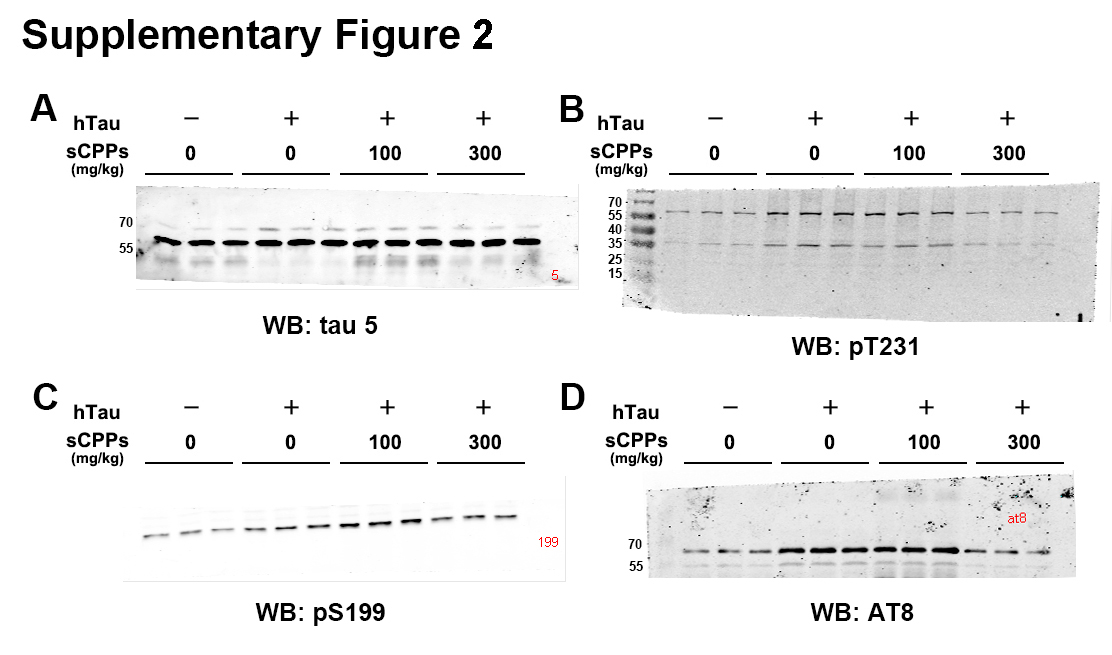

Supplement: FIGURE S2 — The level and phosphorylation tau in AAV2-hTau infected mice. Levels of total tau (A), phosphorylated tau at Ser199 (B), Thr231 (C), and Ser202/Thr205 (AT8) (D) were detected by western blotting in AAV2-hTau infected mice. [file Image_2.JPEG]
