# Supplementary material for: Codonopsis pilosula Polysaccharide Attenuates Tau Hyperphosphorylation and Cognitive Impairments in hTau Infected Mice
Source: Front Mol Neurosci. 2018 Nov 27;11:437. doi: 10.3389/fnmol.2018.00437 (PMC6277749; doi:10.3389/fnmol.2018.00437)
Supplement: TABLE S1 — A recognition index. [file Table_1.DOCX]

**Supplementary Table 1. A Recognition Index**

|  |  | Col. Stats | WT | hTau |
| --- | --- | --- | --- | --- |
| WT | hTau | Number of values | 5 | 5 |
| 0.55263 | 0.46552 |  |  |  |
| 0.46988 | 0.54878 | Minimum | 0.4699 | 0.4655 |
| 0.54545 | 0.48649 | 25% Percentile | 0.4927 | 0.476 |
| 0.53333 | 0.5102 | Median | 0.5333 | 0.5102 |
| 0.51546 | 0.51667 | 75% Percentile | 0.549 | 0.5327 |
|  |  | Maximum | 0.5526 | 0.5488 |
|  |  |  |  |  |
|  |  | Mean | 0.5234 | 0.5055 |
|  |  | Std. Deviation | 0.03304 | 0.03154 |
|  |  | Std. Error | 0.01478 | 0.01411 |
|  |  |  |  |  |
|  |  | Lower 95% CI of mean | 0.4823 | 0.4664 |
|  |  | Upper 95% CI of mean | 0.5644 | 0.5447 |
|  |  |  |  |  |
|  |  | KS normality test |  |  |
|  |  | KS distance | 0.2187 | 0.162 |
|  |  | P value | > 0.10 | > 0.10 |
|  |  | Passed normality test (alpha=0.05)? | Yes | Yes |
|  |  | P value summary | ns | ns |
|  |  |  |  |  |
|  |  | Sum | 2.617 | 2.528 |
