# Supplementary material for: Codonopsis pilosula Polysaccharide Attenuates Tau Hyperphosphorylation and Cognitive Impairments in hTau Infected Mice
Source: Front Mol Neurosci. 2018 Nov 27;11:437. doi: 10.3389/fnmol.2018.00437 (PMC6277749; doi:10.3389/fnmol.2018.00437)
Supplement: TABLE S2 — B recognition index. [file Table_2.DOCX]

**Supplementary Table 2. B Recognition Index**

|  |  | Col. Stats | WT | hTau |
| --- | --- | --- | --- | --- |
| WT | hTau | Number of values | 5 | 5 |
| 0.44737 | 0.53448 |  |  |  |
| 0.53012 | 0.45122 | Minimum | 0.4474 | 0.4512 |
| 0.45455 | 0.51351 | 25% Percentile | 0.451 | 0.4673 |
| 0.46667 | 0.4898 | Median | 0.4667 | 0.4898 |
| 0.48454 | 0.48333 | 75% Percentile | 0.5073 | 0.524 |
|  |  | Maximum | 0.5301 | 0.5345 |
|  |  |  |  |  |
|  |  | Mean | 0.4766 | 0.4945 |
|  |  | Std. Deviation | 0.03304 | 0.03154 |
|  |  | Std. Error | 0.01478 | 0.01411 |
|  |  |  |  |  |
|  |  | Lower 95% CI of mean | 0.4356 | 0.4553 |
|  |  | Upper 95% CI of mean | 0.5177 | 0.5336 |
|  |  |  |  |  |
|  |  | KS normality test |  |  |
|  |  | KS distance | 0.2187 | 0.162 |
|  |  | P value | > 0.10 | > 0.10 |
|  |  | Passed normality test (alpha=0.05)? | Yes | Yes |
|  |  | P value summary | ns | ns |
|  |  |  |  |  |
|  |  | Sum | 2.383 | 2.472 |
