# Supplementary material for: Codonopsis pilosula Polysaccharide Attenuates Tau Hyperphosphorylation and Cognitive Impairments in hTau Infected Mice
Source: Front Mol Neurosci. 2018 Nov 27;11:437. doi: 10.3389/fnmol.2018.00437 (PMC6277749; doi:10.3389/fnmol.2018.00437)
Supplement: TABLE S3 — D recognition index. [file Table_3.DOCX]

**Supplementary Table 3. D Recognition Index**

|  |  | Col. Stats | WT | hTau |
| --- | --- | --- | --- | --- |
| WT | hTau | Number of values | 5 | 5 |
| 0.69748 | 0.54217 |  |  |  |
| 0.71739 | 0.53061 | Minimum | 0.641 | 0.5067 |
| 0.70833 | 0.52809 | 25% Percentile | 0.6693 | 0.5174 |
| 0.64103 | 0.50667 | Median | 0.7083 | 0.5306 |
| 0.72881 | 0.53846 | 75% Percentile | 0.7231 | 0.5403 |
|  |  | Maximum | 0.7288 | 0.5422 |
|  |  |  |  |  |
|  |  | Mean | 0.6986 | 0.5292 |
|  |  | Std. Deviation | 0.03419 | 0.01383 |
|  |  | Std. Error | 0.01529 | 0.00618 |
|  |  |  |  |  |
|  |  | Lower 95% CI of mean | 0.6562 | 0.512 |
|  |  | Upper 95% CI of mean | 0.7411 | 0.5464 |
|  |  |  |  |  |
|  |  | KS normality test |  |  |
|  |  | KS distance | 0.2868 | 0.268 |
|  |  | P value | > 0.10 | > 0.10 |
|  |  | Passed normality test (alpha=0.05)? | Yes | Yes |
|  |  | P value summary | ns | ns |
|  |  |  |  |  |
|  |  | Sum | 3.493 | 2.646 |
