# Supplementary material for: Codonopsis pilosula Polysaccharide Attenuates Tau Hyperphosphorylation and Cognitive Impairments in hTau Infected Mice
Source: Front Mol Neurosci. 2018 Nov 27;11:437. doi: 10.3389/fnmol.2018.00437 (PMC6277749; doi:10.3389/fnmol.2018.00437)
Supplement: TABLE S4 — Discrimination index-24H. [file Table_4.DOCX]

**Supplementary Table 4. Discrimination Index-24H**

|  |  | Col. Stats | WT | hTau |
| --- | --- | --- | --- | --- |
| WT | hTau | Number of values | 5 | 5 |
| 0.39496 | 0.08434 |  |  |  |
| 0.43478 | 0.06122 | Minimum | 0.2821 | 0.01333 |
| 0.41667 | 0.05618 | 25% Percentile | 0.3385 | 0.03476 |
| 0.28205 | 0.01333 | Median | 0.4167 | 0.06122 |
| 0.45763 | 0.07692 | 75% Percentile | 0.4462 | 0.08063 |
|  |  | Maximum | 0.4576 | 0.08434 |
|  |  |  |  |  |
|  |  | Mean | 0.3972 | 0.0584 |
|  |  | Std. Deviation | 0.06839 | 0.02766 |
|  |  | Std. Error | 0.03058 | 0.01237 |
|  |  |  |  |  |
|  |  | Lower 95% CI of mean | 0.3123 | 0.02406 |
|  |  | Upper 95% CI of mean | 0.4821 | 0.09274 |
|  |  |  |  |  |
|  |  | KS normality test |  |  |
|  |  | KS distance | 0.2868 | 0.268 |
|  |  | P value | > 0.10 | > 0.10 |
|  |  | Passed normality test (alpha=0.05)? | Yes | Yes |
|  |  | P value summary | ns | ns |
|  |  |  |  |  |
|  |  | Sum | 1.986 | 0.292 |
